# Dihydromyricetin Reverses Thioacetamide-Induced Liver Fibrosis Through Inhibiting NF-κB-Mediated Inflammation and TGF-β1-Regulated of PI3K/Akt Signaling Pathway

**DOI:** 10.3389/fphar.2021.783886

**Published:** 2021-11-15

**Authors:** Yingchun Zhao, Xinglong Liu, Chuanbo Ding, Yan Gu, Wencong Liu

**Affiliations:** ^1^ College of Chinese Medicinal Materials, Jilin Agricultural University, Changchun, China; ^2^ College of Agriculture, Jilin Agricultural University, Changchun, China; ^3^ National and Local Joint Engineering Research Center for Ginseng Breeding and Development, Changchun, China

**Keywords:** dihydromyricetin, liver fibrosis, NF-κB signaling pathway, inflammation, hepatic stellate cell activation, anti-apoptosis

## Abstract

As a natural active substance, dihydromyricetin (DHM) has been proven to have good hepatoprotective activity. However, the therapeutic effect of DHM on liver fibrosis, which has become a liver disease threatening the health of people around the world, has not been studied to date. The purpose of this study was to investigate the effect of DHM as a new nutritional supplement on thioacetamide (TAA)-induced liver fibrosis. The liver fibrosis model was established by intraperitoneal injection of TAA (200 mg/kg, every 3 days) for 8 weeks, and oral administration of DHM (20 mg/kg and 40 mg/kg, daily) after 4 weeks of TAA-induced liver fibrosis. The results showed that DHM treatment significantly inhibited the activities of alanine aminotransferase (ALT) (37.81 ± 7.62 U/L) and aspartate aminotransferase (AST) (55.18 ± 10.94 U/L) in serum of liver fibrosis mice, and increased the levels of superoxide dismutase (SOD) and glutathione (GSH) while reversed the level of malondialdehyde (MDA). In addition, histopathological examination illustrated that TAA induced the inflammatory infiltration, apoptosis and fibroatherosclerotic deposition in liver, which was further confirmed by western-blot and immunofluorescence staining. Moreover, DHM inhibited hepatocyte apoptosis by regulating the phosphorylation level of phosphatidylinositol 3-kinase (PI3K), protein kinase-B (AKT) and its downstream apoptotic protein family. Interestingly, immunofluorescence staining showed that DHM treatment significantly inhibited alpha smooth muscle actin (α-SMA), which was a marker of hepatic stellate cell activation, and regulated the expression of transforming growth factor (TGF-β1). Importantly, supplementation with DHM significantly inhibited the release of nuclear factor kappa-B (NF-κB) signaling pathway and pro-inflammatory factors in liver tissue induced by TAA, and improved liver fiber diseases, such as tumor necrosis factor alpha (TNF-α) and recombinant rat IL-1β (IL-1β). In conclusion, the evidence of this study revealed that DHM is a potential hepatoprotective and health factor, and which also provides the possibility for the treatment of liver fibrosis.

## Introduction

As an important immune and metabolic organ of the human body, liver is vulnerable to damage from various causes, including hepatitis virus infection, alcohol and drug injury, autoimmune response and metabolic diseases (Marrone et al., 2016). Chronic liver disease and liver fibrosis have always been the focus of contemporary medical research, and people have tried to establish a model of liver fibrosis to adapt to contemporary research needs in recent years (Sepanlou et al., 2020). As a potent hepatotoxin, TAA-induced liver fibrosis is a well-recognised model for the development of liver damage, regenerative nodules and fibrosis, similar to human liver fibrosis (Passos et al., 2010; Nascimento et al., 2018). Numerous experiments have shown that long-term administration of TAA leads to proliferative liver nodules, liver fibrosis, hepatocellular adenomas and hepatocellular carcinoma (Li et al., 2002; Yeh et al., 2004; Constantinou et al., 2007; Jantararussamee et al., 2021). The toxicity of TAA arises from its bioactivation of reactive metabolites, including TAA-sulfur oxides and reactive oxygen species (ROS) *via* the monooxygenases flavin adenine dinucleotide (FAD) and cytochrome P2E1 (CYP2E1) (Tunez et al., 2005). These metabolites bind to cellular macromolecules, induce oxidative stress through lipid peroxidation and increased free radical load, and participate in fibril formation (Chilakapati et al., 2005). More seriously, TAA attacks hepatocyte DNA, RNA and protein-related synthetic enzymes in the body, produces toxic effects, affects liver metabolism, causes metabolic disorders and leads to imbalance of antioxidant defense system, and aggravates chronic liver inflammation and even liver necrosis (Hessien et al., 2010; Kadir et al., 2011). Therefore, TAA induces a progressive liver disease characterised by inflammation, oxidative stress, apoptosis, extensive fibrosis and eventual development of hepatocellular carcinoma, thereby mimicking the progression of inflammation/fibrosis/malignancy in patients.

Studies have shown that the key to prevent liver fibrosis is to inhibit the activation of hepatic stellate cells (HSCs) or promote the apoptosis of HSCs (Zhang et al., 2017a). In addition, some profibrotic mediators are also involved in stimulating HSCs activation and myofibroblast transformation, such as TGF-β1, which is not only involved in the pleiotropic cytokine produced by extracellular matrix (ECM), but also regulated the signaling pathways related to cell proliferation, differentiation and apoptosis in the liver (Gressner and Weiskirchen, 2006; Vaidya and Kale, 2015; Zhang, 2017). Therefore, this may be an important way to study the mechanism and targeted therapy of liver fibrosis at the molecular level in the future by inhibiting the multi effect cytokine and its downstream apoptotic proteins, reducing collagen accumulation and ECM deposition (Bakin et al., 2000). NF-κB pathway participates in regulating a variety of inflammatory cell responses, and has been used for targeted therapy of many inflammatory diseases. Studies have shown that inflammatory cells can further activate HSCs, which may be the main source of myofibroblasts in the liver (Cubero, 2016). NF-κB is involved in the inhibition of apoptosis through the transcriptional induction of a variety of anti-apoptotic factors, including B cell-lymphoma-2 (Bcl-2) family proteins (Zheng et al., 2009). In addition, the expression of NF-κB-regulated gene products is also involved in the apoptotic Bcl-2 family (Bui et al., 2001; Gupta et al., 2002). Therefore, this may be a crucial way to improve TAA-induced liver fibrosis by reducing NF-κB-mediated inflammation and apoptotic signaling pathways.

Vine tea (*Ampelopsis grossedentata*) is a traditional medicinal plant widely used to improve health or suppressing disease in Chinese folk (Ye et al., 2015). In addition to being used as a tea drink, vine tea was traditionally used as a folk medicine according to ancient texts for the treatment of fever and cough, stab wounds, bruises, jaundiced hepatitis and sore throats (Gao et al., 2009). Moreover, extracts from vine tea have been proved to have significant anti-inflammatory properties *in vitro* and *in vivo*, and have been recommended as a potential therapeutic agent for inflammation-related diseases (Chen et al., 2015). Importantly, the leaves and stems of vine tea are rich in a large number of natural active substance dihydromyricetin, which has become an important plant resource for the development and research of functional products (Gao et al., 2009; Kou and Chen, 2012; Zhao et al., 2013; Zheng et al., 2014; Ye et al., 2015; Zhang et al., 2020; Carneiro et al., 2021). It is worth mentioning that as a natural active substance, dihydromyricetin from vine tea has been approved by the Food and Drug Administration (FDA) as a nutritional supplement and can be added in food industry.

The hepatoprotective effects of dihydromyricetin are now well recognized. zhuangwei Zhang et al. investigated the down-regulation of the Akt/Bad pathway in HepG2 cells to reduce apoptosis (Zhang et al., 2017b). Sijing Dong et al. used dihydromyricetin to counteract acetaminophen-induced liver injury by modulating lipid homeostasis, cell death and regenerative pathways (Dong et al., 2019). Yi Zeng et al. demonstrated a protective effect of dihydromyricetin in improving non-alcoholic steatohepatitis (Zeng et al., 2020). Xi Zhou et al. demonstrated that dihydromyricetin attenuated carbon tetrachloride-induced liver injury by modulating autophagy and inhibiting the activation of hepatic stellate cells (Zhou et al., 2021). Ping Qiu et al. also demonstrated the protective effect of dihydromyricetin against ethanol-induced liver injury (Qiu et al., 2017). However, dihydromyricetin has not been found to improve liver fibrosis. Based on this, the present study aimed to explore the improvement effect of dihydromyricetin on liver fibrosis by establishing a mouse model of TAA-induced liver fibrosis and further revealed its possible molecular mechanism, supplemented the relevant research and provided a potential clinical possibility for the treatment of liver disease.

## Materials and Methods

### Chemicals and Reagents

DHM (purity ≥99.0%) and TAA (purity ≥99.0%) were purchased from Sigma. Hematoxylin-eosin (H and E), TUNEL and Masson stain kits, and commercial assay kits for ALT and AST, SOD, GSH, MDA were obtained from the Nanjing Jiancheng Bioengineering Research Institute (Nanjing, China). The immunofluorescent staining kits and the enhanced chemiluminescence (ECL) kit were purchased from Beyotime science and technology Co., Ltd. (Beijing, China). The antibody of rabbit monoclonal α-SMA, TGF-β1, CYP2E1, Cleaved Caspase-3, Caspase3, Cleaved Caspase-9, Caspase-9, IκB kinase α (IKK-α), IκB kinase β (IKK-β), *p*-IKKα, *p*-IKKβ, inhibitor of IκBα (IκBα), *p*-IκBα, NF-κB, p-NF-κB, PI3K, p-PI3K, Akt, *p*-Akt, B-associated X (Bax), Bcl-2, β-actin and secondary antibodies for western blot were all obtained from Abcam (Cambridge, United Kingdom).

### Animals and Drug Treatment

The forty fully-grown male ICR mice (4–6 weeks old, weight 20–25 g) were purchased from Changchun Yisi Experimental Animal Co., Ltd. (Changchun, China), and the certificate of quality was No. SCXK (JI)-2019–0,001. Mice were maintained under a professional animal breeding room with constant temperature and humidity and pathogen-free, and unlimited access to sufficient food and water. This study was approved by the animal research ethics committee of Jilin Agricultural University, ethics approval No.: 2019–08–28–002.

**FIGURE 1:**
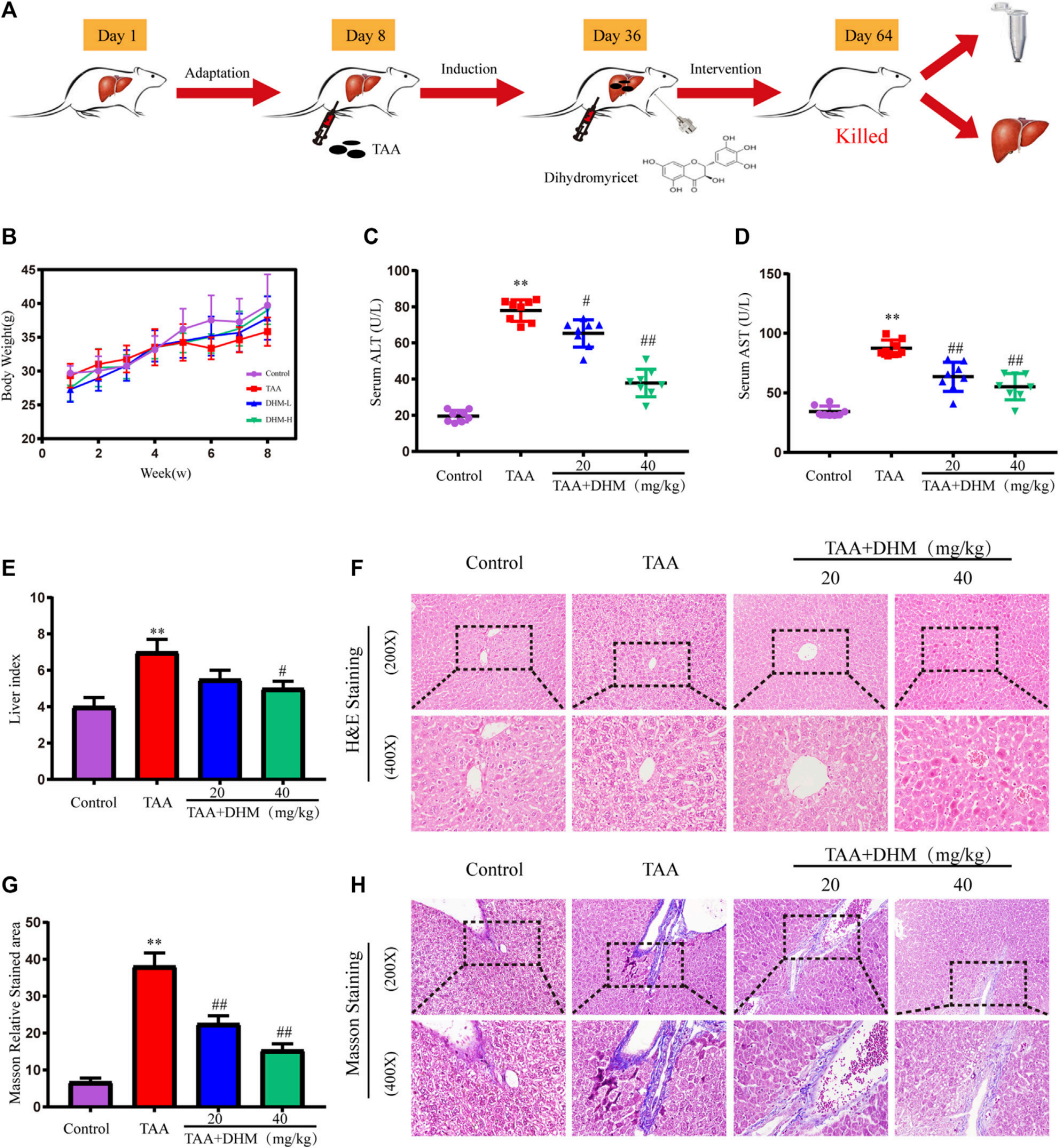
DHM ameliorated TAA-induced hepatic injury in mice. The experimental design **(A)**; Body weight **(B)** and serum levels of ALT **(C)** and AST **(D)** (*n* = 8); liver index **(E)**, H&E staining **(F)**; Masson relative stained area **(G)** and Masson’ strichrome staining **(H)**; Data was expressed as the mean ± SD. The differences between groups were analyzed by two tailed T-test or one-way analysis of variance (ANOVA). **p < 0.01, *p < 0.05 *versus* control group; ##*p* < 0.01, #*p* < 0.05 *versus* TAA group.

Forty mice were divided into four groups under 7 days adaptive feeding conditions (control, TAA group, TAA + DHM 20 mg/kg and 40 mg/kg groups, *n* = 10), and the detailed experimental design process was shown in Figure 1A. All mice (excluding the control group) were received TAA (200 mg/kg, every 3 days) from day 8 to day 64. Moreover, the TAA + DHM groups were received the treatment of DHM (20 mg/kg and 40 mg/kg, daily) from day 36 to day 64. At the completion of the last TAA injection, mice were treated with a 12-h fast. After anaesthetics treatment with 2% chloral hydrate intraperitoneally, blood samples were collected from the eyes of mice and serum was collected by centrifugation (4°C, 3,500 rmp, 10 min). After the mice were killed after cervical spondylectomy, the livers were collected immediately, measured and the liver index of the mice was obtained by the formula (liver weight/body weight) × 100. Some of them were soaked in 4% paraformaldehyde solution, and the others were stored at −80°C.

### Determination of Biochemical Indicators

ALT and AST are involved in central metabolism in the body, and the level of ALT and AST transaminases are markers of liver injury. Therefore, we evaluated the degree of liver injury by detecting the levels of serum ALT and AST. Serum ALT and AST levels were measured using commercial kits purchased from Nanjing Jiancheng reagent company. Determine the absorbance according to the instructions and calculate the ALT and AST levels according to the formula.

### Histopathological Staining

The liver tissue samples were stained by H&E routine staining, and evaluated by histopathological according to standard procedures. In order to evaluate the degree of fibrosis in liver samples, the connective tissue, muscle fibers and collagen fibers in liver tissues were stained with Masson trichrome staining kit. Observe pathological changes such as fatty degeneration, necrosis, and inflammatory cell infiltration of liver cells under an optical microscope.

### Determination of Oxidative Stress Indicators

The levels of GSH, MDA, and SOD are important indicators reflecting oxidative stress damage (Ding et al., 2021). Therefore, the levels of GSH, MDA and SOD in liver tissue were detected. Take an appropriate amount of liver tissue and fully grind it with 9 times the volume of normal saline under low temperature to make 10% tissue homogenate. The operation was carried out according to the method of each kit and finally the absorbance was measured and calculated according to the formula.

### Immunofluorescence Staining

In order to assess the expression of apoptosis and inflammation-related proteins in liver tissue induced by TAA, immunofluorescence staining was performed on liver tissue sections of each group (Hou et al., 2015). After the tissue sections were deparaffinized with xylene solution and hydrated in ethanol, the antigen was recovered by microwave in a citrate buffer solution (0.01 M, pH 6.0) for 20 min. After washing 3 times with PBS, the tissue was incubated with 1% bovine serum albumin (BSA) for 1 h, and then the primary antibody caspase 3 (1:200), CYP2E1 (1:200), TNF-α (1:400) was added dropwise, TGF-β1 (1:200) and α-SMA (1:200) were incubated overnight at 4°C. The next day, it was labeled with a fluorescent secondary antibody at 37°C for 30 min and then labeled with SABC-CY3 (1:100) again. The nucleus was stained with 4,6 diamidino-2-phenylindole (DAPI). The protein expression was observed with a fluorescence microscope, and the immunofluorescence intensity was analyzed with Image-Pro Plus 6.0 software.

### Western Blot Analysis

RIPA lysate lyses liver tissue and extracts its protein. The extracted protein samples were separated by 10% SDS-PAGE gel electrophoresis, and then transferred to PVDF membrane. After blocking with skim milk for 1.5 h, then use primary antibodies (Bax, Bcl-2, recombinant human B-cell Leukemia/Lymphoma XL (Bcl-XL), cysteine proteases3 (Caspases3), cleaved caspase 3, cysteine proteases9 (caspase 9), cleaved caspase 9, PI3K, p-PI3K, AKT, *p*-AKT, IKKα/β, *p*-IKKα/β, IκBα, *p*-IκBα, NF-κB (p65), p-NF-κB (p-p65), TNF-α, IL-1β, β-actin at 4°C Incubate overnight. Then, the membrane was washed 3 times in Tris-buffered saline (TBS) containing 0.1% Tween-20, and then the membrane was incubated with the secondary antibody for 1 h at room temperature. After the band was detected by ECL luminescent solution, the protein expression was displayed by Bio-Lad Laboratories Segrate.

### TUNEL Staining

TUNEL staining was used to analyze the extent of apoptosis in hepatocyte nuclei. Liver tissue antigen was extracted with 20 μg/ml proteinase K for 10 min, then TUNEL reaction mixture (TdT:dUTP, 1:9) was added for 1 h in the dark at 37°C. The cells were washed three times with PBS and then observed under a fluorescent microscope for apoptosis.

### Statistical Analysis

Statistical analysis was carried out using SPSS statistical software. The differences between groups were analyzed by two tailed T-test or one-way analysis of variance (ANOVA). The results are shown as the mean ± SD, and the significance level is defined as *p* < 0.05. Graphs were drawed with GraphPad Prism software (version 7).

## Results

### Dihydromyricetin Alleviates Thioacetamide-Induced Chronic Hepatic Injury

During the experiment, the weight of mice in each group were recorded. Although there was no significant difference in body weight, the overall increase trend of body weight was lower than that of the control group in TAA-treated mice (Figure 1B). Besides, a significant increase in relative liver index was observed in the TAA model group compared to the control group (Figure 1E), with the increase in liver mass attributed to the accumulation of fat and breakdown of liver tissue. However, the DHM intervention resulted in a reduction in liver index. Furthermore, the appropriate commercial kits were used for serological examinations, and the levels of ALT and AST in serum were measured to determine the severity of liver injury. Compared with the control group, the levels of ALT and AST in the serum of mice were significantly increased by TAA-induced (*p* < 0.01, Figures 1C,D). Nevertheless, ALT and AST levels were reduced to varying degrees by DHM supplementation, with a particularly significant improvement in DHM (40 mg/kg), suggesting that DHM significantly improved TAA-induced hepatotoxicity.

### Dihydromyricetin Improve Thioacetamide-Induced Liver Histopathological Damage

In order to evaluate the hepatoprotective effect of DHM, the improvement effect of DHM on TAA-induced liver dysfunction was examined by histopathology in this study. H and E staining showed that the hepatocytes in the control group were arranged regularly, with large round nucleoli and uniform cytoplasm, and there was no inflammatory cell infiltration in the portal area (Figure 1F). However, the liver structure treated with TAA was disordered, the liver lobule structure was destroyed or disappeared, the hepatocytes were arranged irregularly and had obvious apoptosis, and the fibrous deposition around the central vein gradually increased, and a large number of inflammatory cells and fibroblasts were infiltrated in the fibrous septum. In contrast, continuous treatment with DHM significantly alleviated pathological changes by TAA-induced, lipid degeneration and inflammatory injury in liver.

In addition, the results observed by Masson staining were basically consistent with the fibrosis in H and E staining (Figure 1H). The structure of central vein and catheter endothelial fibers in the control group was normal, and there was no abnormal fiber proliferation in other places, but there was slight inflammation. However, a large number of collagen fibers were observed in the portal area and central vein of the liver tissue treated with TAA, the hepatic lobules were divided by different fiber intervals, and there were obvious fiber adhesion and a large number of inflammatory cells between portal duct and central vein. Obviously, DHM treatment significantly inhibited the production of collagen fibers compared with the TAA group, there were a small amount of scattered proliferative fibers in hepatic lobules, and inflammatory cells were significantly reduced. In particular, the results of collagen area measurement by software also demonstrated that DHM treatment reduced collagen deposition and resulted in a reduction in collagen area (Figure 1G).

### Dihydromyricetin Alleviated Thioacetamide-Induced Hepatotoxicity

**FIGURE 2:**
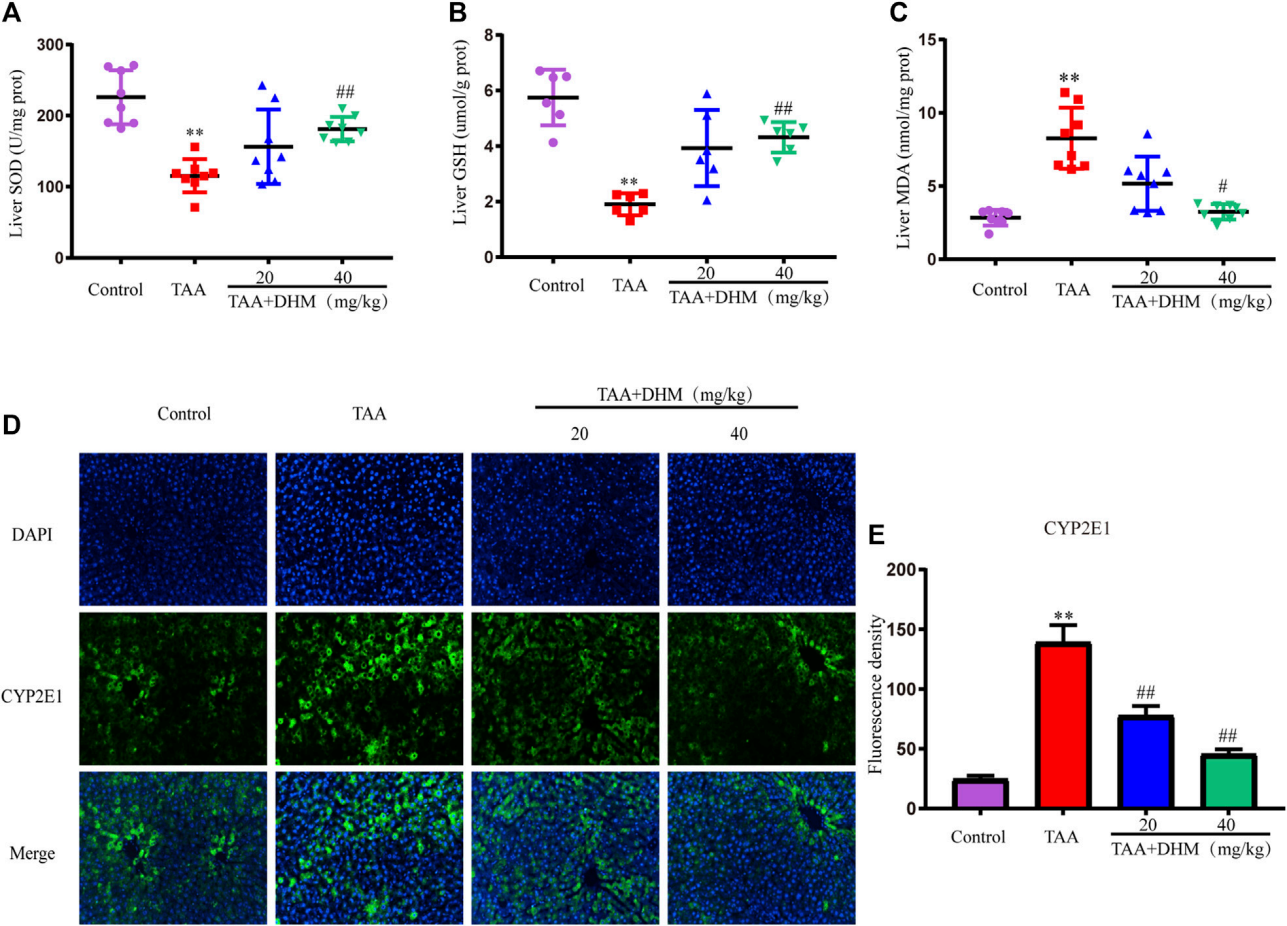
DHM attenuates TAA-induced oxidative stress and hepatotoxicity. The hepatic levels of SOD **(E)**, GSH **(F)** and MDA **(G)** in TAA-induced liver fibrosis (*n* = 6–8); The protein expression levels of CYP2E1 **(E)** and Fluorescence density **(F)**; Data was expressed as the mean ± SD. The differences between groups were analyzed by two tailed T-test or one-way analysis of variance (ANOVA). **p < 0.01, *p < 0.05 *versus* control group; ##*p* < 0.01, #*p* < 0.05 *versus* TAA group.

Studies have shown that oxidative stress damage was related to TAA-induced liver toxicity (Chen et al., 2021). To evaluate the effect of DHM on TAA-induced oxidative stress in the liver, oxidative stress parameters were measured in the liver, such as SOD, GSH, and MDA. TAA induction significantly increased MDA levels but decreased SOD activity, accompanied by GSH depletion, compared to the control group (*p* < 0.01, Figures 2A–C). On the contrary, DHM-treated significantly increased the activities of GSH and SOD, while decreased the level of MDA in a dose-dependent manner (*p* < 0.05). The above results indicated that DHM significantly reduced TAA-induced oxidative stress damage.

Since the CYP-mediated biological activity plays an important role in TAA-induced hepatotoxicity, in order to further confirm the damage and hepatotoxicity caused by TAA to the liver, the expression of CYP2E1 protein was examined by immunofluorescence staining in liver tissue. The results showed that TAA caused the overexpression of CYP2E1 metabolic enzymes, while treatment with DHM significantly reduced the expression of CYP2E1 (*p* < 0.01, Figures 2D,E). These results also indicated to a certain extent that DHM improved TAA-induced oxidative stress damage and hepatotoxicity, especially in the DHM (40 mg/kg) group, which had a significant ameliorative effect and approached that of the control group.

**FIGURE 3:**
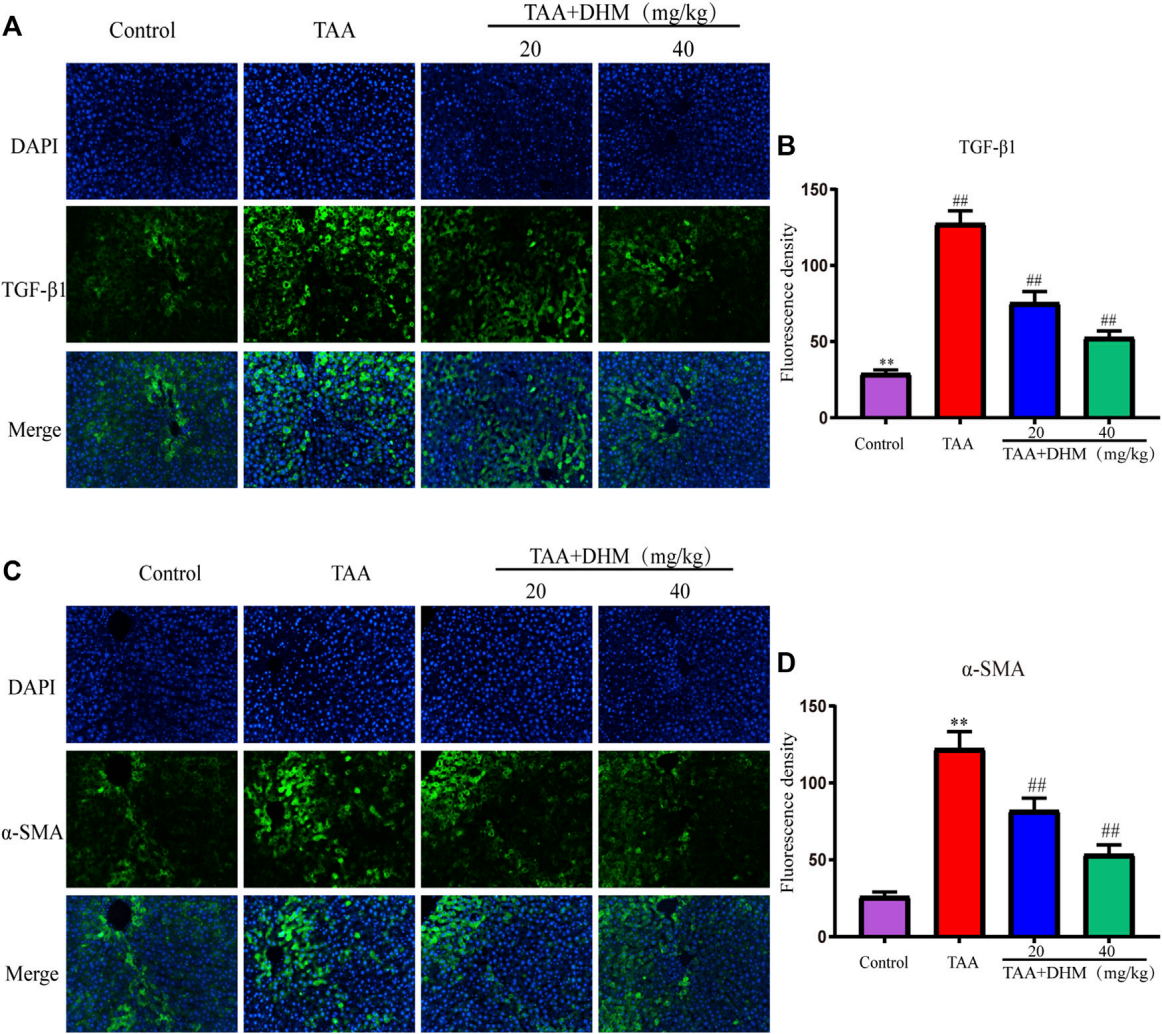
DHM alleviated TAA-induced activation of hepatic stellate cells. The protein expression levels of TGF-β1 **(A)** and Fluorescence density **(B)**, The protein expression levels of α-SMA **(C)** and Fluorescence density **(D)**; Data was expressed as the mean ± SD. The differences between groups were analyzed by two tailed T-test or one-way analysis of variance (ANOVA). **p < 0.01, *p < 0.05 *versus* control group; ##*p* < 0.01, #*p* < 0.05 *versus* TAA group.

The activation of HSCs is a key event leading to liver fibrosis, and the HSCs activation marker α-SMA and pro-fibrotic factor TGF-β1 in liver were analyzed by immunofluorescence staining in this study. The results demonstrated that the positive expression and fluorescence intensity of TGF-β1 and α- SMA were significantly improved by immunofluorescence staining compared with the control group after long-term TAA induction (*p* < 0.01, Figures 3A–D). On the contrary, the administration of DHM (20, 40 mg/kg) effectively reduced the secretion of TGF-β1 and the expression of α-SMA, which intuitively indicated that DHM inhibited the activation of HSCs and thus reversed the development of liver fibrosis by TAA-induced.

### Dihydromyricetin Attenuates Liver Fibrosis by Inhibiting Thioacetamide-Induced Inflammatory Injury

**FIGURE 4:**
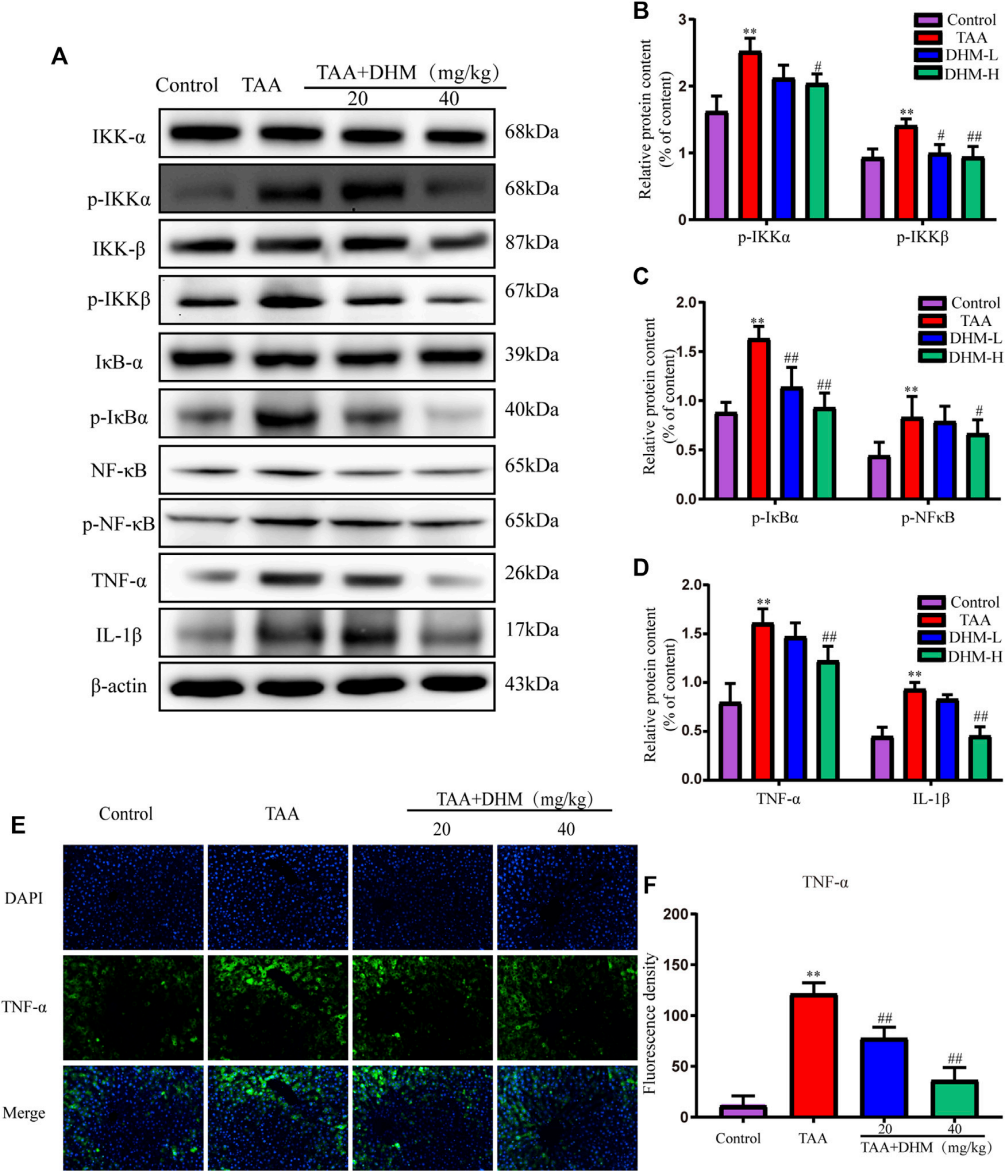
DHM inhibits NF-κB-mediated inflammatory infiltration to reverse TAA-induced liver fibrosis. The protein expression levels of *p*-IKKα, IKKα, IKKβ, p- IKKβ, IκBα, p- IκBα, NFκB, p- NFκB, TNF-α and IL-1β in liver tissues were examined by western blot **(A)**, The protein expression levels of *p*-IKKα, IKKβ **(B)**, The protein expression levels of p- IκBα, p- NFκB **(C)**, The protein expression levels of TNF-α and IL-1β **(D)**, The protein expression levels of TNF-α **(E)** and Fluorescence density **(F)**; Column charts showed relative expression levels of target protein, and protein expression levels were performed by quantification of relative protein expression analysis in each group. Data was expressed as the mean ± SD. The differences between groups were analyzed by two tailed T-test or one-way analysis of variance (ANOVA). **p < 0.01, *p < 0.05 *versus* control group; ##*p* < 0.01, #*p* < 0.05 *versus* TAA group.

In order to explore the anti-inflammatory and hepatoprotective functions of DHM, the expression of NF-κB-related proteins were explored by western blotting in this study. Phosphorylated protein and its upstream inflammatory regulators *p*-IKKα/β and *p*-IκBα were significantly increased by TAA-induced (Figures 4A–D). Interestingly, the expression of NF-κB and its related proteins were significantly reduced after DHM treatment, especially DHM (40 mg/kg) had a significant ameliorating effect (*p* < 0.05). In addition, DHM inhibited the expression of pro-inflammatory factors TNF-α and IL-1β in the liver (*p* < 0.01), which was further confirmed by TNF-α immunofluorescence staining (*p* < 0.01, Figures 4E,F). These evidences suggested that DHM alleviated TAA-induced liver fibrosis by inhibiting the expression of NF-κB signaling pathway and other inflammatory factors.

### Dihydromyricetin Decreases Hepatocyte Apoptosis by Regulating the PI3K/Akt Signaling Pathway

**FIGURE 5:**
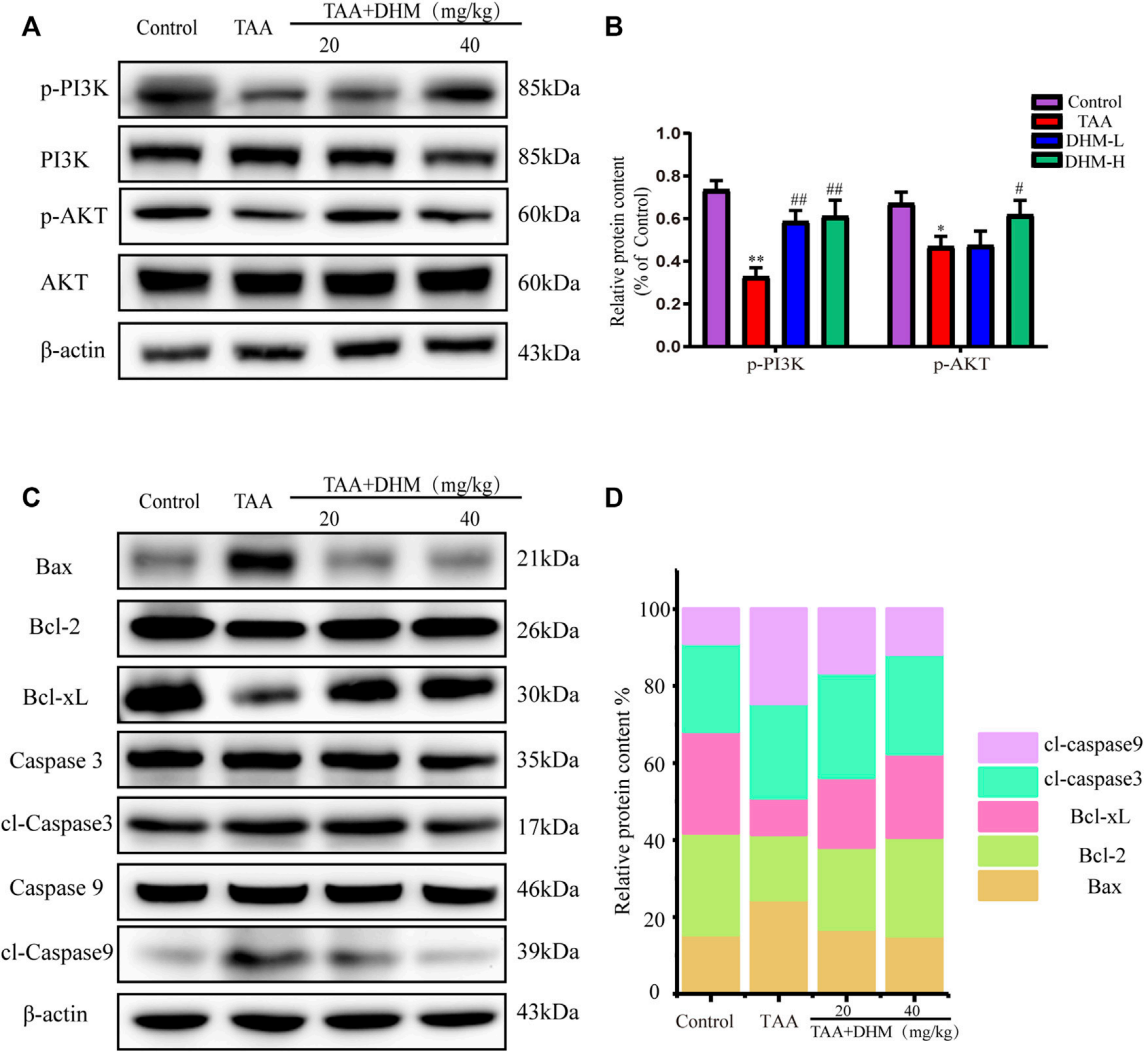
DHM ameliorates PI3K/Akt-mediated apoptosis to reverse TAA-induced hepatic fibrosis. The protein expression levels of p-PI3K, PI3K, AKT, *p*-AKT in liver tissues were examined by western blot. **(A)** and the protein expression levels of p-PI3K and *p*-AKT protein **(B)**; The protein expression levels of Bax, Bcl-2, Bcl-xL, *p*-Caspase 3, Caspase 3, *p*-Caspase 9, Caspase 9 in liver tissues were examined by western blot **(C)** and the protein expression levels of Bax, Bcl-2, Bcl-xL, *p*-Caspase 3 and *p*-Caspase 9 protein **(D)**; β-actin protein was used as a loading control. Column charts showed relative expression levels of target protein, and protein expression levels were performed by quantification of relative protein expression analysis in each group. Data was expressed as the mean ± SD. The differences between groups were analyzed by two tailed T-test or one-way analysis of variance (ANOVA). **p < 0.01, *p < 0.05 *versus* control group; ##*p* < 0.01, #*p* < 0.05 *versus* TAA group.

**FIGURE 6:**
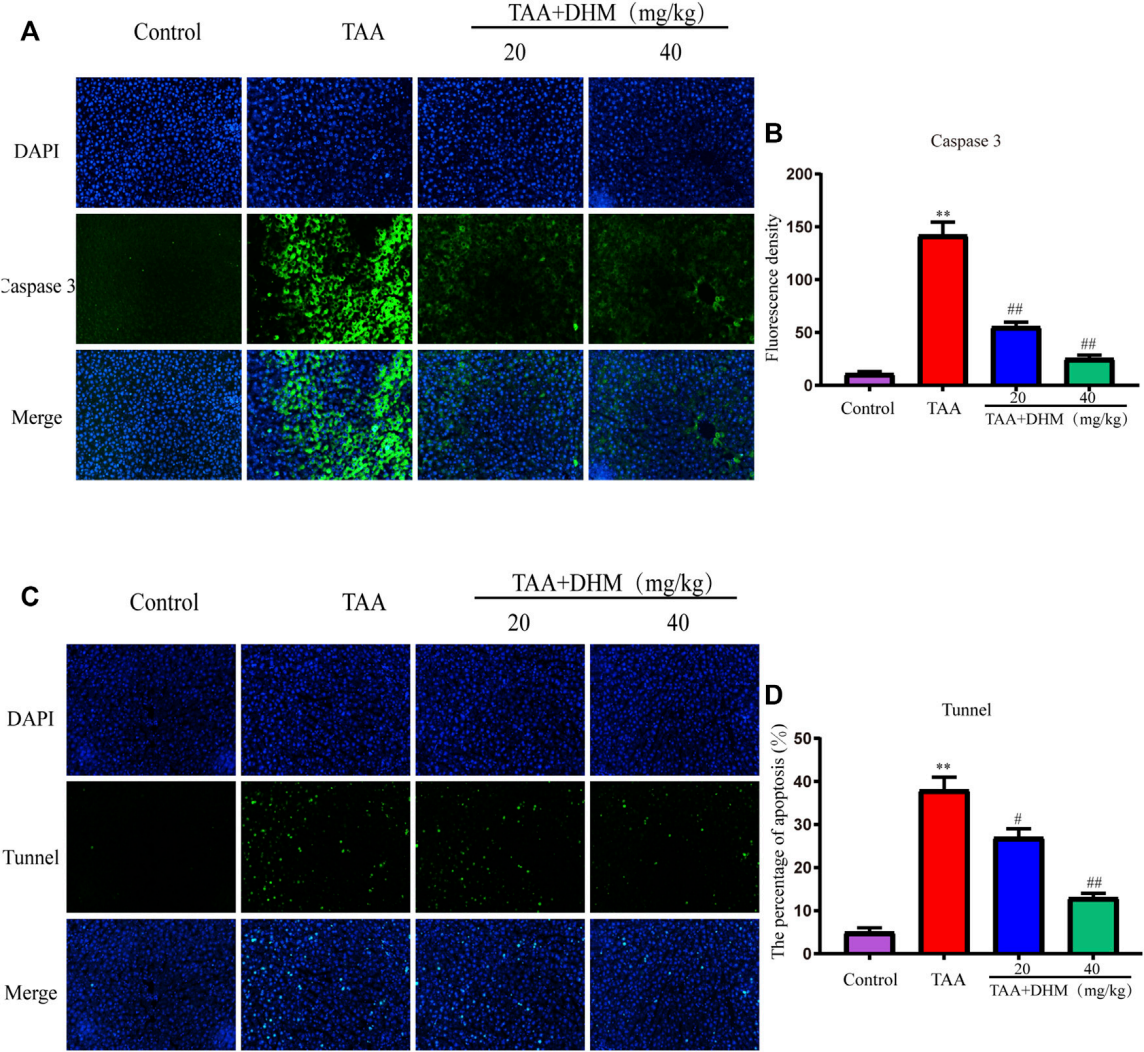
DHM alleviated TAA-induced apoptosis of hepatocytes. The protein expression levels of caspase 3 **(A)** and Fluorescence density **(B)**, Fluorescence analysis of hepatocyte apoptosis **(C)** and Fluorescence density **(D)**; Data was expressed as the mean ± SD. The differences between groups were analyzed by two tailed T-test or one-way analysis of variance (ANOVA). **p < 0.01, *p < 0.05 *versus* control group; ##*p* < 0.01, #*p* < 0.05 *versus* TAA group.

To further explore the molecular mechanism of DHM antagonizing TAA-induced liver fibrosis, western blotting and immunofluorescence staining were used to analyze the expression level of PI3K/Akt signaling pathway, including its downstream the expression levels of Bcl-2 family and caspase family proteins, which was also an important way to regulate the proliferation, apoptosis and differentiation of activated HSCs. The results observed that the expression of p-PI3K and *p*-AKT proteins were significantly down-regulated after TAA treatment (*p* < 0.05, Figures 5A,B). On the contrary, DHM increased the expression of PI3K/AKT proteins in liver tissue. Not only that, DHM pretreatment also significantly reduced the expression of caspase pathway related proteins. Apparently, the expression of the pro-apoptotic proteins Bax, cleaved Caspase-9 and cleaved Caspase-3 were increased significantly after TAA induction, while the expression of anti-apoptotic proteins Bcl-2 and Bcl-XL were significantly decreased in this study (*p* < 0.01, Figures 5C,D). However, these proteins were effectively reversed after DHM treatment. In addition, the immunofluorescence staining of Caspase 3 was analyzed, and Caspase 3 positive cells may be the key event of activated HSCs (Mi et al., 2019). Interestingly, the results of caspase 3 obtained by immunofluorescence analysis were consistent with western blot analysis, which showed that DHM exhibited anti-apoptotic effects in TAA-induced chronic liver fibrosis and further promoted the apoptosis of activated HSCs (Figures 6A,B).

TUNEL staining was used to identify hepatocyte apoptosis and the number of green apoptotic nuclei was significantly increased after TAA establishment of the model compared to the control, indicating that TAA caused severe hepatocyte apoptosis. Specifically, DHM (40 mg/kg) administration reduced the amount of green fluorescence, resulting in reduced hepatic apoptosis (*p* < 0.01, Figures 6C,D). These results indicate that DHM has a significant anti-apoptotic effect and is directly proportional to the dose.

## Discussion

This study established the liver fibrosis model of mice by long-term intraperitoneal injection of TAA, and the ameliorative effect of dihydromyricetin on TAA-induced liver fibrosis was observed. In liver disease, transaminase levels in serum are elevated due to damage to liver cells (Williams and Hoofnagle, 1988; Nascimento et al., 2018). Elevated serum aminotransferase levels are a marker of liver injury, therefore we measured ALT and AST in mouse serum to assess the extent of liver injury. Interestingly, the results showed that TAA significantly increased serum ALT and AST concentrations, which was consistent with the results of some researchers (Tsai et al., 2021), while that DHM was able to modify this phenomenon (Salama et al., 2013; Abood et al., 2020; Elnfarawy et al., 2020). In addition, liver indices were measured in mice and the results represent the effect of DHM with hepatoprotective activity on the liver weight/body weight ratio, which was consistent with previous studies (Salama et al., 2013; Abood et al., 2020; Elnfarawy et al., 2020). Under TAA induction, liver tissue is usually fibrotic, leading to an increase in the liver weight ratio, which subsequently decreased after administration of DHM.

Current research generally believes that the activation of HSCs is considered to be a key event in the development of liver fibrosis (Kisseleva and Brenner, 2006; Tsuchida and Friedman, 2017). α-SMA is a marker of HSCs activation, and TGF-β1 is generally considered to be the main hepatic fibrosis-promoting cytokine, and many factors leading to liver disease are related to TGF-β1 in varying degrees (Dewidar et al., 2015). In this study, immunofluorescence staining demonstrated that the positive expression of a-SMA and TGF-β1 were significantly increased in liver tissue by TAA-induced, which was consistent with previous studies (Mi et al., 2019; Zhou et al., 2021). The increase in α-SMA and TGF-β1 in turn induced the synthesis and secretion of ECM by HSC, thus aggravating liver fibrosis (Crosas-Molist et al., 2015). When there is an imbalance between the production and degradation of ECM, it will cause accumulation and scar reaction, and eventually lead to liver collagen deposition and fibrosis (El Taghdouini and van Grunsven, 2016). Furthermore, the histopathological examination of H&E and Masson found that TAA caused a large number of macrophage inflammatory infiltration and liver tissue structural disorders, which speculated that the inflammatory infiltration caused a large number of hepatocyte apoptosis, resulting in the expansion of hepatic veins and sinuses. Besides, the staining results showed that the massive deposition of collagen fibers and portal vein bridging led to the appearance of long fibrous septum, suggesting that long-term TAA induced severe liver fibrosis, while DHM treatment significantly reversed these changes, this was consistent with previous studies (Park et al., 2015; Zeng et al., 2020).

There was a view that many chronic liver diseases were accompanied by the certain degree of oxidative stress, and the extent of hepatic oxidative stress represented the level of liver injury (Uchida et al., 2020). Studies have shown that lipid peroxide products in liver fibrosis may play an important role in the activation of HSCs, especially in the early stage of liver fibrosis (Poli, 2000). Particularly, the excessive accumulation of typical liver lipid peroxidation product reactive oxygen species (ROS) may destroy the intracellular balance, lead to oxidative stress and mitochondrial dysfunction, and further cause the damage of enzyme and non-enzyme defense systems, which in turn causes cell damage (Demarquoy and Borgne, 2015; Horn and Jaiswal, 2018). Among them, these enzymes can transfer superoxide free radicals to other metabolites (Naqshbandi et al., 2013), including SOD and GSH. In this study, the activities of SOD and GSH were significantly reduced, while the level of MDA, as a marker of oxidative stress, was significantly increased by TAA-induced. On the contrary, DHM effectively inhibited the consumption of the above two enzymes and reduced the level of MDA, which was very important for maintaining cell function (Wright et al., 1981; Karadeniz et al., 2011). Consistent with the previous results, DHM had a significant regulatory effect on the decrease of GSH and SOD levels and the increase of MDA levels by TAA-induced.

In addition, studies have shown that inflammatory damage is an important factor leading to many chronic liver diseases, and which is usually accompanied by the development of liver fibrosis, cirrhosis and hepatocellular carcinoma (Koyama and Brenner, 2017). Among them, NF-κB induces the expression of pro-inflammatory cytokines, such as TNF-α and IL-1β, which produces the cytotoxic environment, thereby further triggering the development of chronic inflammation and progressive liver fibrosis (Wang et al., 2008). Therefore, this study detected the inflammation-related proteins in the liver tissue by TAA-induced, including IKKα/β, *p*-IKKα/β, IκBα, *p*-IκBα, NF-κB (p65), p-NF-κB (p-p65), TNF-α, IL-1β, which proved that DHM effectively inhibited the expression of these proteins, thereby reducing the inflammatory injury of liver fibrosis by TAA-induced.

Explicitly, chronic liver disease by TAA-induced causes severe cell injury and apoptosis. Some studies have shown that TAA attacks DNA, RNA and protein synthase in hepatocytes, produce toxic effects, which also induces liver metabolic disorder and even hepatocyte apoptosis. As one of the important signal transduction pathways in cells, PI3K/Akt signaling pathway plays a key role in inhibiting apoptosis and promoting proliferation by affecting a variety of downstream effector molecules related to apoptosis, which is closely related to the occurrence and development of liver diseases. When PI3K binds to growth factor receptors (such as EGFR), it can change the protein structure of AKT, which is a downstream protein of PI3K, and activates or inhibits a series of downstream substrates by phosphorylation, such as the apoptosis related proteins Bad and Caspase9, and further triggers apoptosis (Lin et al., 2015; Fang et al., 2019). Caspases are the key mediator of programmed cell death or apoptosis, and also the guide of the path of living cells to death (Singh et al., 2013). Therefore, the Caspases pathway, as a marker of apoptosis, plays an important role in mediating the process of cell apoptosis (Rehman et al., 2013; Prokhorova et al., 2018). In this study, immunofluorescence and western blot proved that DHM played an anti-apoptotic effect by regulating PI3K/AKT and its downstream pathway caspase-related proteins.

Indeed, the expression of Bcl-2 and Bax apoptotic molecules is also driven by nuclear NF-κB and oxidative stress in addition to PI3K/Akt (Zhang Q. et al., 2017; Sun et al., 2020). NF-κB, an important transcription factor in the nucleus, is not only involved in immunity, inflammation, tissue damage repair and embryonic development processes, but also upregulates the expression levels of inflammatory factors including TNF-α and IL-1β as well as apoptotic genes of the Bcl-2 family (Shou et al., 2002). Akt activates IκB kinase (IKKα/β), causes degradation of the NF-κB inhibitor IκB, results in the release of NF-κB from the cytoplasm for nuclear translocation, activates of its target genes and promotes of cell survival (Bava et al., 2011). In this study, DHM inhibited Bax expression while promoted Bcl-2 expression in TAA-induced liver fibrosis in mice. Bax and Bcl-2 apoptosis-related genes play an important role in the regulation of mitochondria-dependent pathways. In addition, Akt inhibited the activity of proteolytic enzyme caspase 9 and its downstream factor caspase 3 and reduced the activation of apoptosis cascade, which indicated that DHM improved hepatocyte metabolism by regulating hepatocyte proliferation and apoptosis.

**FIGURE 7:**
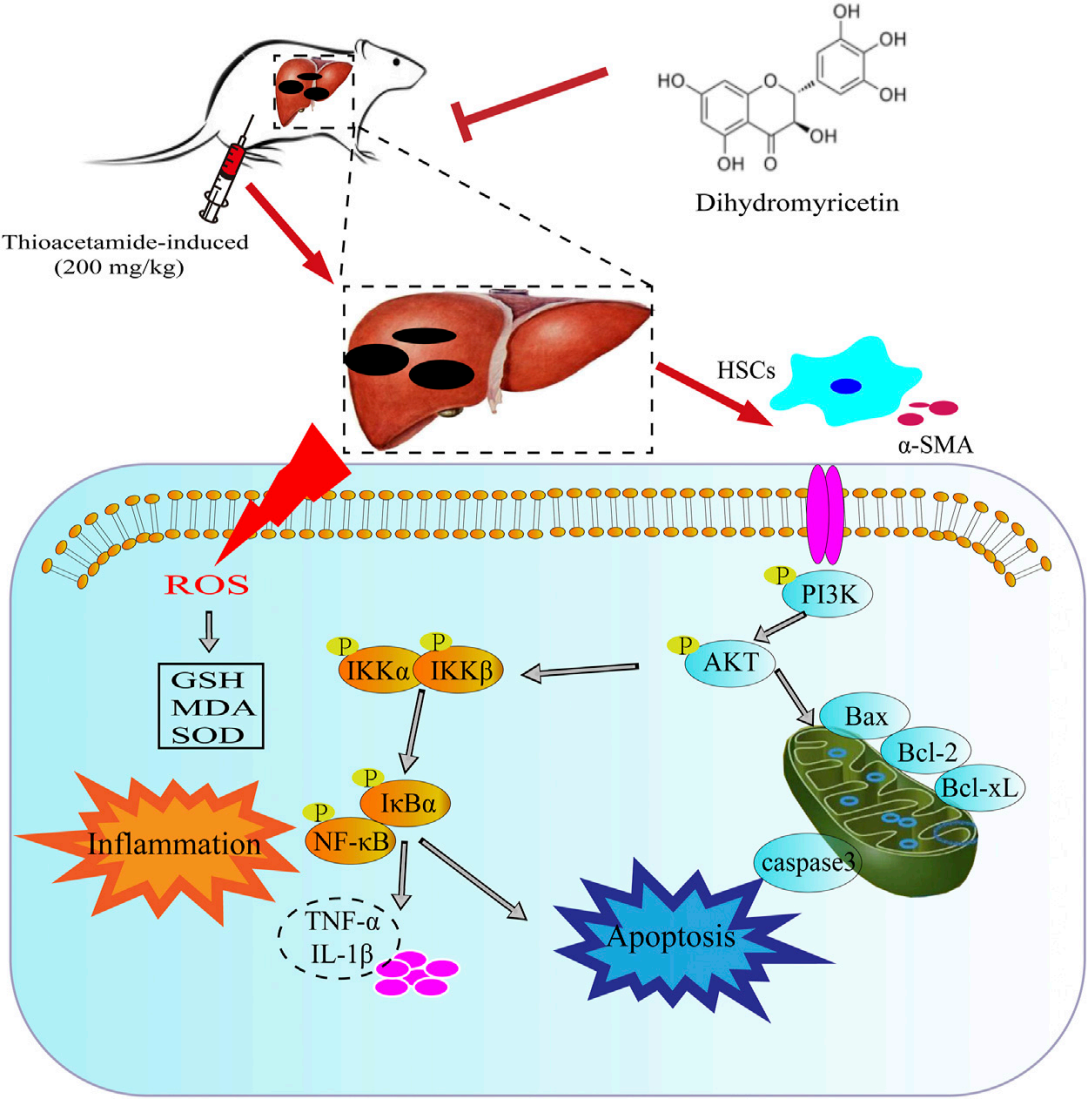
The potential mechanism of DHM in alleviating TAA-induced liver fibrosis. DHM reverses TAA-induced liver fibrosis by inhibiting suppression of HSC activation, NF-κB-mediated inflammatory infiltration and improving PI3K/Akt-mediated apoptosis, which may be a potential mechanism for the hepatoprotective effects of DHM.

In conclusion, the current study clearly demonstrated that TAA administration increased inflammation, oxidative stress, pro-fibrotic markers and hepatotoxic markers in mice and decreased liver function and abnormal liver histology. However, DHM treatment improved damaged liver structure, effectively reduced oxidative stress and hepatotoxic markers. More importantly, DHM reversed TAA-induced liver fibrosis by inhibiting NF-κB-mediated inflammation and TGF-β1-regulated apoptotic proteins downstream of the PI3K/Akt signalling pathway, which also indicated that DHM may be a potential active substance against chronic liver disease (Figure 7).

## Data Availability

The original contributions presented in the study are included in the article/Supplementary Material, further inquiries can be directed to the corresponding authors.

## Glossary

DHM: dihydromyricetin
TAA: thioacetamide
ALT: alanine aminotransferase
AST: aspartate aminotransferase
SOD: superoxide dismutase
GSH: glutathione
MDA: malondialdehyde
PI3K: phosphatidylinositol 3-kinase
AKT: protein kinase B
α-SMA: α-smooth muscle actin
TGF-β1: transforming growth factor-β1
NF-κB: nuclear factor kappa-B
TNF-α: tumor necrosis factor alpha
IL-1β: recombinant rat IL-1β
ROS: reactive oxygen species
FAD: flavin adenine dinucleotide
CYP2E1: cytochrome P2E1
HSCs: hepatic stellate cells
ECM: extracellular matrix
Bcl-2: B cell lymphoma/lewkmia-2
Bcl-XL: recombinant Human B-Cell Leukemia/Lymphoma XL
Bax: Bcl2-associated X
caspase 3: cysteine-dependent aspartate-specifc proteases
Caspases3: cysteine proteases3
caspase 9: cysteine proteases9
NAFLD: non-alcoholic fatty liver disease
H and E: hematoxylin-eosin
ECL: enhanced chemiluminescence
IKK-α: IκB kinase α
IKK-β: IκB kinase β
IκBα: inhibitor of IκBα
Bax: B-associated X
BSA: bovine serum albumin
DAPI: 4,6 diamidino-2-phenylindole
TBS: Tris-buffered saline
CCl4: carbon tetrachloride
IL-6: interleukin-6
